# A Giant Ovarian Mucinous Cystadenoma in an Adolescent Patient: A Case Report

**DOI:** 10.7759/cureus.117751

**Published:** 2026-10-10

**Authors:** Marco Fabricio Bombón Caizaluisa, Antonio Jurado Bambino, Abraham Jurado Rodriguez, Carla Blacio Beltran, Cristian Santillan Coello, Luis Ordoñez Gonzalez

**Affiliations:** 1 Department of Oncological Surgery, Sociedad de Lucha Contra el Cáncer (SOLCA)-Matriz, Guayaquil, ECU; 2 Postgraduate Program in General Surgery, Universidad de Especialidades Espíritu Santo, Guayaquil, ECU; 3 Department of Oncology and Surgery, Universidad de Guayaquil, Guayaquil, ECU; 4 Postgraduate Program in General Surgery, Universidad de Especailidades Espíritu Santo, Guayaquil, ECU; 5 Department of Surgical Oncology and Breast Surgery, Consultorio Hospital de Especialidades Borja Clinic, Guayaquil, ECU; 6 Department of General Surgery, Hospital Monte Sinai, Guayaquil, ECU

**Keywords:** adolescent, benign ovarian tumor, mucinous ovarian cystadenoma, salpingo-oophorectomy, surgery

## Abstract

We present the case of a 14-year-old female patient with no significant medical history who presented with long-standing abdominal pain and distension caused by a giant abdominal mass. Physical examination revealed a distended abdomen that was tender to both superficial and deep palpation, with an enormous mass extending from the pelvis to the epigastric region. Additional tests were performed, including laboratory tests, abdominal ultrasound, and oral contrast-enhanced CT of the abdomen and pelvis, which revealed an intracavitary mass occupying the entire pelvis and part of the upper abdomen, measuring 24 x 13 x 32 cm. The patient underwent exploratory laparotomy, right salpingo-oophorectomy, and peritoneal lavage. The histopathological report revealed a benign mucinous cystadenoma, with peritoneal lavage negative for malignancy. Postoperative recovery was uneventful, and the patient has remained asymptomatic during subsequent clinical follow-up.

Given the low incidence of this neoplasm in our setting, this case highlights the importance of a timely approach and management based on current scientific evidence. It is important to note that surgical treatment in the early stages allows for total resection of the lesion, with low recurrence rates.

## Introduction

Ovarian tumors are conditions that occur primarily during the second decade of childhood, and most are germ cell tumors [1,2].

The causes of ovarian enlargement, both in childhood and adolescence, include conditions such as functional cysts, ovarian torsion, and malignant or benign neoplasms [3]. Most epithelial ovarian tumors are cystadenomas; mucinous types are less common than serous ones, and the vast majority are benign [4]. Almost all cases of mucinous ovarian cystadenomas typically present with abdominal distension and pain attributed to a large cystic mass that grows progressively and gradually [5]. The analysis of tumor markers, along with radiological studies, is an important and essential tool in the diagnosis of both malignant and benign ovarian pathologies [6]. Elevated serum levels of tumor markers are generally associated with malignant conditions [1,7]. However, microscopic examination is of great diagnostic value, as it typically reveals architectural and cytological atypia, including benign, borderline, and carcinomatous areas [8].

Typically, a combination of the patient’s medical history, physical examination findings, imaging data, and tumor marker profile is used to distinguish malignant ovarian masses from benign ones [9]. In this context, a radiological evaluation of the tumor’s size and complexity is essential for stratifying the risk of malignancy prior to histopathological evaluation [10].

The gold standard for this type of pathology is surgical treatment. The goal of therapeutic surgery is the radical and complete excision of the mass without rupture, preserving normal ovarian tissue whenever possible [11].

This case report describes the clinical presentation, diagnosis, and treatment initiated following histopathological confirmation of a mucinous ovarian cystadenoma, taking into account the latest evidence to guide diagnosis and specific management.

## Case presentation

A 14-year-old female adolescent with no significant personal or family medical history, who began menstruating at age 10 and recently had her first sexual intercourse, presented to this health center with a large abdominal mass of unknown origin causing abdominal distension and moderate abdominal pain, which had been present for several months and had worsened in recent weeks, accompanied by sporadic metrorrhagia. She had bowel movements but denied nausea or vomiting. Physical examination revealed a distended abdomen, tender to superficial and deep palpation, and tympanic on percussion; a massive mass extending from the pelvis to the epigastric region is palpable.

Additional tests, including laboratory tests, abdominal ultrasound, and oral contrast-enhanced pelvic and abdominal CT scans, were ordered. The results of paraclinical studies are presented in Table 1.

**Table 1:** Clinical laboratory tests on admission

Parameter	Result	Reference range
White blood cell count (WBC)	11.28 10^3/u	5,000-10,000/µL
Neutrophils	72%	37-72%
Hemoglobin	11.6 g/dL	12-16 g/dL
Hematocrit	35.6 %	36- 44%
Platelet count	286 10^3/uL	150,000-450,000/µL
Serum sodium	135	135-145 mmol/L
Serum potassium	4.50	3.5-5.1 mmol/L
Serum chlorine	103	98-106 mmol/L
Carcinoembryonic antigen	3.16	3-5 ng/mL
Human chorionic gonadotropin (hCG)	Negative	
CA 19.9	16.22	0-37 U/ml
CA 125	30	<35 U/ml
HE4	25	24- 60.5 pmol/L
Risk of Ovarian Malignancy Algorithm (ROMA) Index	Low	

The abdominal ultrasound revealed a massive cystic mass extending from the pelvis to the epigastric region, likely originating in the right ovary.

The abdominal and pelvic CT scan (oral contrast) reported an extensive intracavitary mass occupying the entire pelvis and part of the upper abdomen, measuring approximately 32 x 24 x 13 cm, consistent with a cystic tumor of adnexal origin (Figures 1-2).

**Figure 1:**
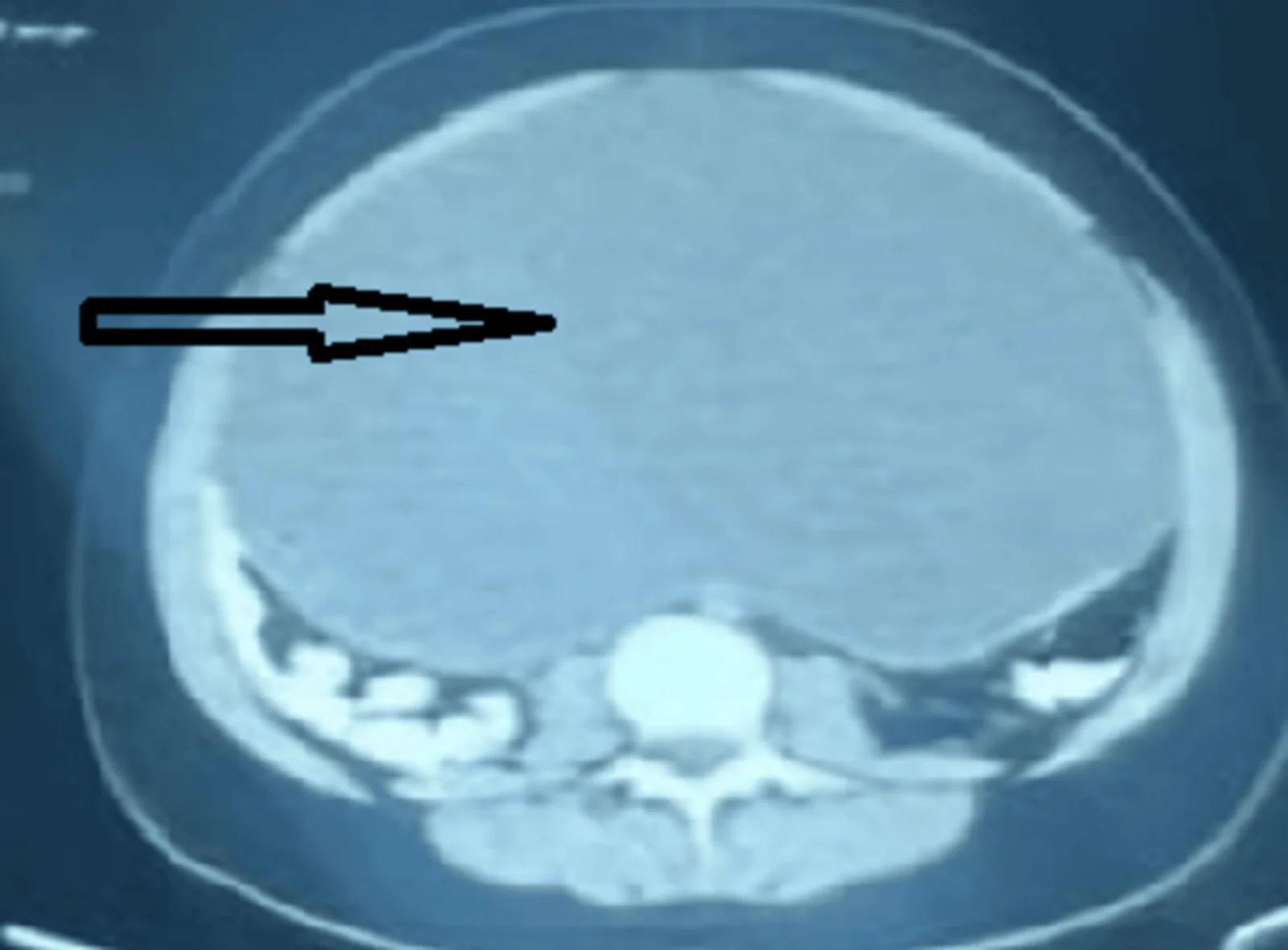
Axial CT scan of the abdomen and pelvis without contrast showing an intracavitary mass occupying the entire pelvis and part of the upper abdomen, measuring approximately 32 x 24 x 13 cm.

**Figure 2:**
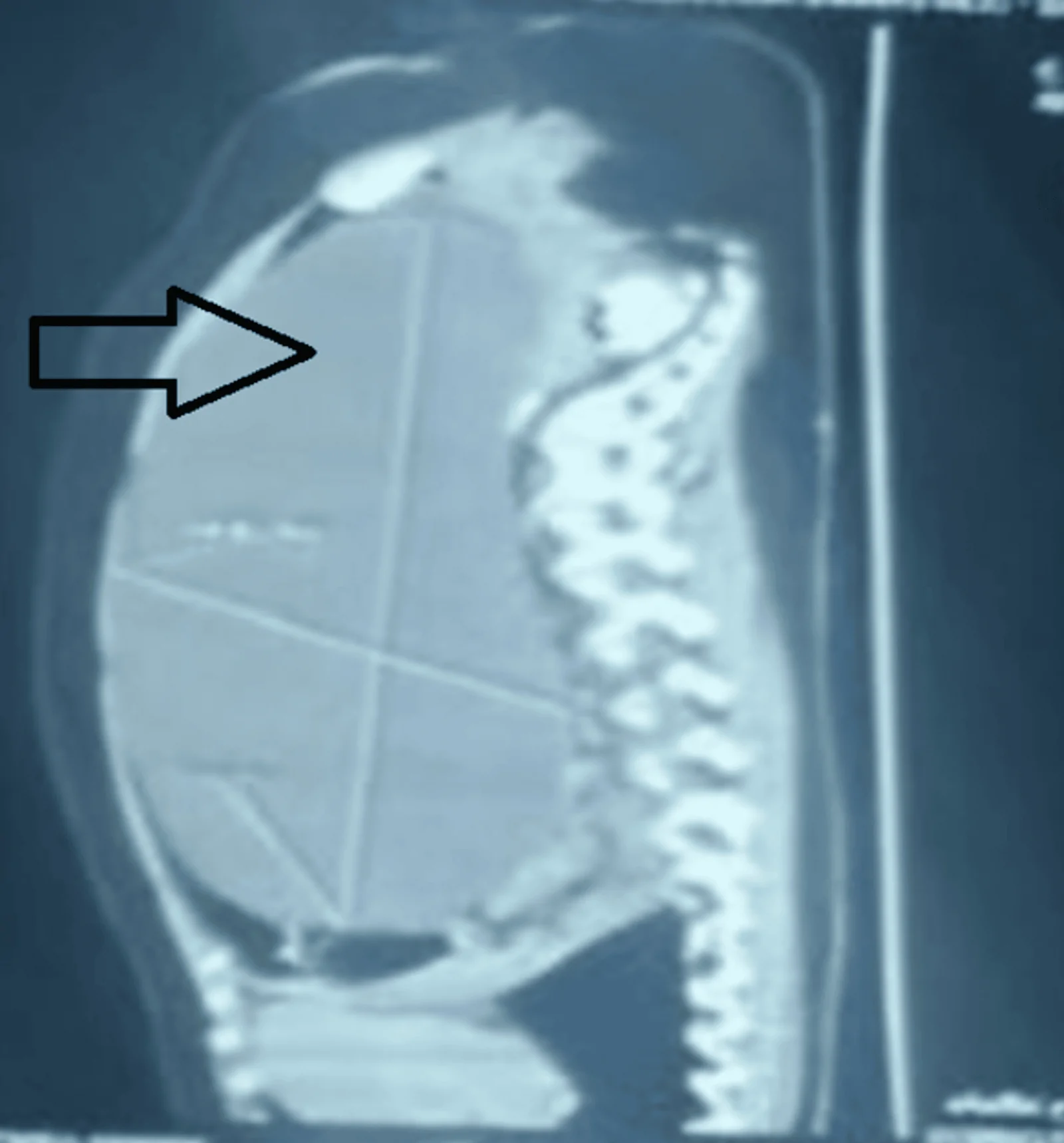
Sagittal CT scan of the abdomen and pelvis without contrast showing an intracavitary mass occupying the entire pelvis and part of the upper abdomen, measuring approximately 32 x 24 x 13 cm.

The patient underwent surgery under general anesthesia. An exploratory laparotomy, right salpingo-oophorectomy, and peritoneal lavage were done. Intraoperatively, a tumor of the right adnexa weighing 7,720 g, measuring 35 × 24 × 11.4 cm, with an intact surface that was pink and pale (Figure 3), was found.

**Figure 3:**
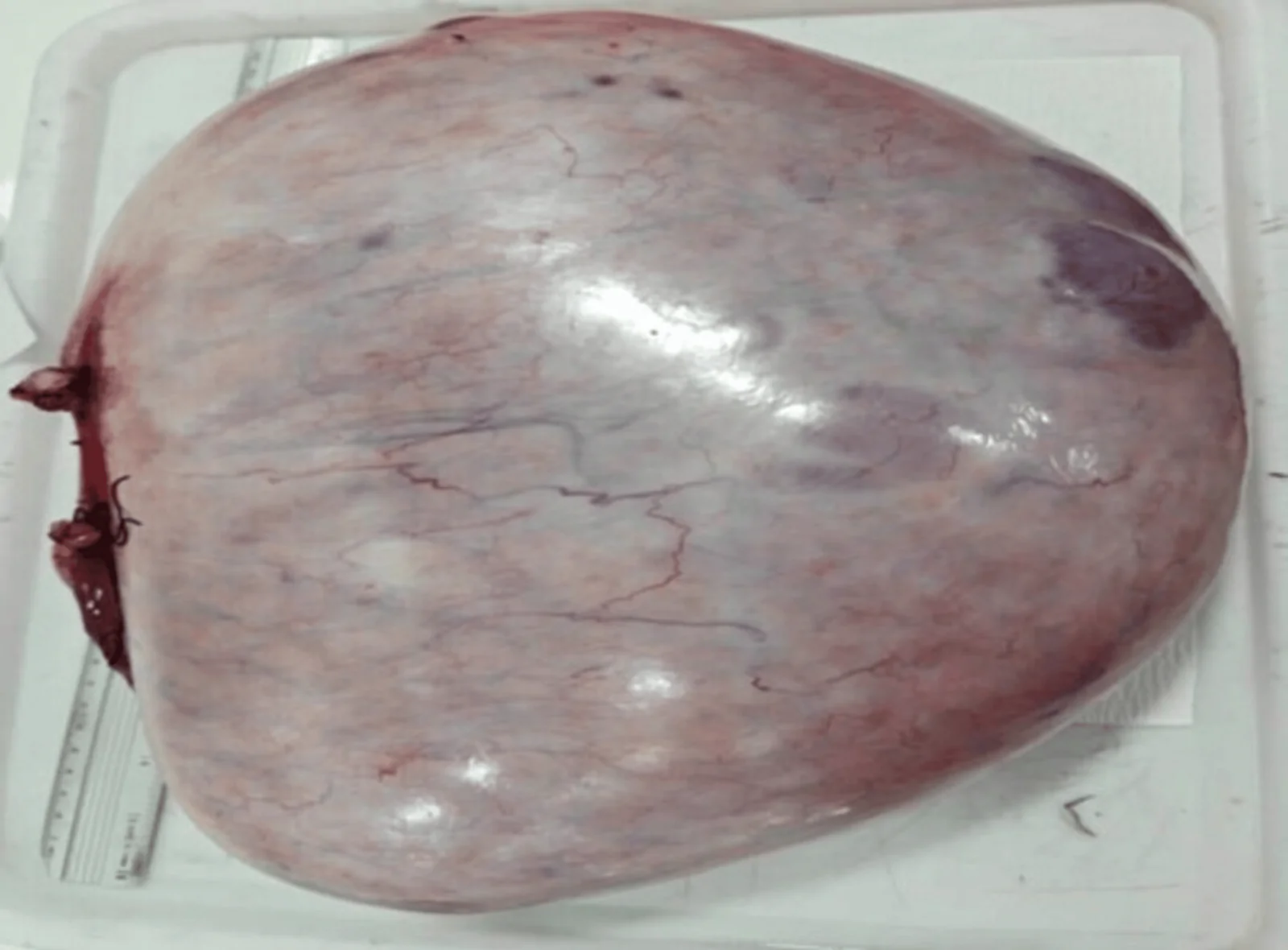
A tumor attached to the right adnexa, measuring 35 x 24 x 11.4 cm, intact in its entirety was found.

A small amount of inflammatory fluid, approximately 50 ml, was found. The procedure was completed without complications, with minimal bleeding. The patient was discharged two days after surgery; she was able to tolerate food, had normal bowel movements, and had an abdominal drain that showed a decrease in serosanguineous discharge, which was subsequently removed without complications during an outpatient visit.

The histopathological diagnosis reported a mucinous tumor largely corresponding to a benign mucinous cystadenoma. The fallopian tube was benign. Peritoneal washings were negative for malignancy. No immunohistochemical or mutational studies were performed, given the benign nature of the pathology specimens and the absence of diagnostic uncertainty.

During subsequent follow-up visits, to ensure strict monitoring and follow-up, a new plain abdominal and pelvic CT scan with intravenous contrast was performed, which showed an adequate postoperative course, with no evidence of tumor recurrence, ascites, or retroperitoneal lymphadenopathy.

Currently, one year after surgery, the patient continues to undergo periodic follow-up visits in the Department of Surgical Oncology. She did not receive adjuvant therapy because the tumor was benign. She has maintained an adequate clinical course, with no palpable mass on physical examination; the patient has a normal appetite and performs her daily activities without difficulty.

## Discussion

Pediatric ovarian neoplasms are relatively rare, with an estimated incidence of 2.2 to 2.6 cases per 100,000 pediatric girls and adolescents [1,2]. Of these neoplasms, most are benign, and the reported range of malignant cases is only 10% to 27%. The most commonly recognized types in this group are mature cystic teratomas, mucinous cystadenomas, and serous cystadenomas [2,3].

Mucinous cystadenomas are quite rare and uncommon in this age group [2]. Therefore, due to their low prevalence, there is little data on their epidemiology, management, and specific treatment; the evidence reviewed is primarily extrapolated from other histological tumor types and case reports [3].

Genetic studies of this condition have identified KRAS mutations in up to 60% of benign tumors [4]. However, there may be other mutations that play an important role in tumorigenesis. In this context, mutations in P53, CDKN2A, SMAD4, BRAF, BRCA1, PTEN, PIK3CA, AATK, and RNF43 have been reported [5].

The clinical presentation consists of abdominal pain, bloating, and occasionally menstrual irregularities, with or without a palpable pelvic mass [4,5]; of these symptoms, abdominal pain (57%) and a palpable pelvic mass (46%) are the most common [5]. The adolescent described in this particular case presented with a large palpable pelvic mass, which caused moderate abdominal pain accompanied by a small amount of sporadic bloody vaginal discharge. In this context, according to the reviewed evidence, approximately 21% of patients with epithelial ovarian tumors are asymptomatic [4,5], which means that symptoms are often not detected until the disease has progressed [6].

Although most pediatric ovarian masses are benign, early diagnosis is important to prevent complications such as ovarian torsion or rupture and, most importantly, to rule out malignant conditions [1,6]. Transabdominal ultrasound is the initial imaging technique used to evaluate abdominal masses in the pediatric population and in adolescents. MRI or CT are complementary techniques used for a more comprehensive evaluation, to determine the differential diagnosis, and to guide therapeutic decision-making [7]. With regard to laboratory tests and tumor markers, CA 125 has been widely used as a marker for epithelial ovarian tumors. However, its positive predictive value is currently debatable [7]. Although serum CA 125 levels (> 35 U/ml) are elevated in most patients with epithelial ovarian carcinoma, this value is also elevated in approximately 1% of non-neoplastic conditions such as endometriosis, cirrhosis, pancreatitis, pelvic inflammatory disease, and advanced non-ovarian abdominal malignancies [1,8].

Therefore, CA-125 is rarely used as a single parameter in predicting malignancy, as it is supplemented by the introduction of HE4, which, according to the evidence reviewed, demonstrates greater sensitivity and specificity than CA-125; however, the true value lies in the simultaneous use of both tumor markers. In addition, the Risk of Ovarian Malignancy Algorithm (ROMA) index allows for estimating the probability that a mass or tumor in the ovary is malignant [9].

In our patient, both serum CA-125 and HE4 levels were within normal ranges, indicating a low ROMA index for ovarian cancer. It is important to note the specific recommendation to perform an analysis of ovarian tumor markers prior to surgery to aid in intraoperative management and the definitive pathological diagnosis [10].

Therefore, these findings, taken together with the intraoperative characteristics and the histopathological result, led to a diagnosis of benign mucinous cystadenoma.

The currently recommended treatment is typically unilateral oophorectomy, salpingo-oophorectomy, or cystectomy [1,11]. If healthy ovarian tissue is identified and preservation is feasible, it should be preserved [12]. The size of the tumor does not always allow for surgery to preserve the affected ovary; however, several studies in adolescents have shown that this treatment option was safe and appropriate, with no evidence of recurrence [12]. In this context, most of the literature suggests that the risk of recurrence in a contralateral or even ipsilateral adnexa is very low, and no deaths related to benign mucinous cystadenomas have been reported [13].

In our adolescent patient, the tumor was very large, and it was not possible to identify the ovary or the ipsilateral fallopian tube, which appeared normal; therefore, a salpingo-oophorectomy with total excision was performed without rupture of the lesion.

## Conclusions

Mucinous ovarian cystadenoma is a rare benign condition in girls and adolescents, with a low global incidence, that can grow to a large size. Early and timely diagnosis, management, and treatment allow for complete resection of the tumor, resulting in lower rates of complications and recurrence. This particular case has had a favorable clinical course following surgical treatment, with no current clinical or imaging evidence of tumor recurrence.
